# Synthesis, characterization and evaluation of transfection efficiency of dexamethasone conjugated poly(propyleneimine) nanocarriers for gene delivery

**DOI:** 10.1080/13880209.2018.1517183

**Published:** 2018-09-30

**Authors:** Bizhan Malaekeh-Nikouei, Mehdi Rezaee, Leila Gholami, Naghmeh Sanjar Mousavi, Reza Kazemi Oskuee

**Affiliations:** a Nanotechnology Research Center, Institute of Pharmaceutical Technology, Mashhad University of Medical Sciences, Mashhad, Iran;; b Department of Medical Biotechnology, Faculty of Medicine, Mashhad University of Medical Sciences, Mashhad, Iran;; c Student Research Committee, Mashhad University of Medical Sciences, Mashhad, Iran;; d Neurogenic Inflammation Research Center, Mashhad University of Medical Sciences, Mashhad, Iran;; e Targeted Drug Delivery Research Center, Institute of Pharmaceutical Technology, Mashhad University of Medical Sciences, Mashhad, Iran

**Keywords:** Gene transfer techniques, non-viral vectors, dendrimers, polyplexes, nuclear localization signals

## Abstract

**Context:** Polypropylenimine (PPI), a cationic dendrimer with defined structure and positive surface charge, is a potent non-viral vector. Dexamethasone (Dexa) conveys to the nucleus through interaction with its intracellular receptor.

**Objective:** This study develops efficient and non-toxic gene carriers through conjugation of Dexa at various percentages (5, 10 and 20%) to the fourth and the fifth generation PPIs (PPIG4s and PPIG5s).

**Materials and methods:** The 21-OH group of Dexa (0.536 mmol) was modified with methanesulfonyl chloride (0.644 mmol) to activate it (Dexa-mesylate), and then it was conjugated to PPIs using Traut's reagent. After dialysis (48 h) and lyophilization, the physicochemical characteristics of products (PPI-Dexa) including zeta potential, size, buffering capacity and DNA condensing capability were investigated and compared with unmodified PPIs. Moreover, the cytotoxicity and transfection activity of the Dexa-modified PPIs were assessed using Neuro2A cells.

**Results:** Transfection of PPIG4 was close to PEI 25 kDa. Although the addition of Dexa to PPIG4s did not improve their transfection, their cytotoxicity was improved; especially in the carrier to DNA weight ratios (C/P) of one and two. The Dexa conjugation to PPIG5s enhanced their transfection at C/P ratio of one in both 5% (1.3-fold) and 10% (1.6-fold) Dexa grafting, of which the best result was observed in PPIG5-Dexa 10% at C/P ratio of one.

**Discussion and conclusions:** The modification of PPIs with Dexa is a promising approach to improve their cytotoxicity and transfection. The higher optimization of physicochemical characteristics, the better cell transfection and toxicity will be achieved.

## Introduction

With multiple potential targets for the treatment of various diseases, gene therapy becomes an interesting field of research for scientists to find novel therapies for various diseases (Thomas et al. 2003; Li and Huang 2006). Different types of therapeutic nucleic acids (NAs) including plasmid DNA (pDNA), oligodeoxynucleotides (ODNs), RNA interference (RNAi), microRNAs (miR) have been studied for the treatment of various disorders, but these molecules are prone to degradation in a biological environment and their cellular uptake is inefficient. In this regard, various types of NA carriers have been developed which are principally categorized into viral and non-viral vectors (Krützfeldt 2016; Pahle and Walther 2016; Rezaee et al. 2016). Due mainly to their safety and capacity to rationally being designed, non-viral vectors were vastly investigated and considered as alternatives to the highly potent but immunogenic viral vectors (Thomas et al. 2003).

Dendrimers, being tailor-made to be purposefully designed, have been considered as efficient carriers for pharmaceuticals as well as polynucleotide molecules (Dufès et al. 2005; Alavi et al. 2017). Owing to their defined molecular structure and variable functional groups located on their outer surfaces and inner moieties, dendrimers are proper platforms for the development of biopharmaceuticals as well as nanomedicine carriers (Inoue 2000). Polypropylenimine (PPI), a cationic dendrimer with a high ratio of the positively-charged functional groups on its surface to its volume, forms a nano-sized vector in complex with NAs (Frechet 1994; Russ et al. 2008). With the branched tree-like molecular architecture, the higher generation PPIs provide numerous terminal groups which could be modified with different adducts (Mammen et al. 1998; Moghadam Ariaee et al. 2016).

Three crucial steps (barriers) of efficient gene delivery are cellular uptake, endosomal release and nuclear translocation. The nuclear transportation of pDNA is one of the main obstacles of transgene expression because pDNA should enter the nucleus for transcription (Ma et al. 2010; Wang et al. 2012). Many approaches have been developed for nucleus transportation, one of which is the incorporation of some adducts which act as nuclear localization signals (NLSs) (Lange et al. 2007). Glucocorticoids (GCs), a group of pharmaceuticals with anti-inflammatory effects, have intracellular receptors and could efficiently inter the nucleus through steroid-mediated gene delivery mechanism (Rebuffat et al. 2001). The potency of nuclear translocation of GCs is mainly depended on their affinity to glucocorticoid receptors (GRs). The higher their affinity to GRs, the more nuclear translocation would occur (Ma et al. 2009; Malaekeh-Nikouei et al. 2017). A synthetic and highly potent GC, dexamethasone (Dexa), binds to its intracellular receptor and this complex actively conveys to the nucleus through nuclear pore complex which has been dilated to 60 nm (Shahin et al. 2005; Mi Bae et al. 2007). Thus, the cargoes attached to Dexa would actively be passed to the nucleus.

Therefore, this study was aimed to design and develop an efficient non-viral gene carrier based on PPI dendrimer with two different generations G4 and G5, conjugated with various grafting percentages of Dexa, and characterize them as well as evaluating their cell transfection and toxicity.

## Materials and methods

### Materials

PPI G4 and PPI G5 were purchased from SyMO-Chem BV (Eindhoven, the Netherlands). Dexa was supplied by Aburaihan Pharmaceutical Co (Tehran, Iran). Hydrochloric acid, sodium hydroxide, ethyl acetate, dimethyl sulfoxide (DMSO) and anhydrous pyridine were purchased from Merck (Darmstadt, Germany). 2,4,6-Trinitrobenzene sulfonic acid (TNBS), sodium tetraborate (Na_2_B_4_O_7_·10H_2_O) (borax), methanesulfonyl chloride and ethidium bromide (EtBr) were purchased from Sigma-Aldrich (Darmstadt, Germany). Dulbecco's Modified Eagle Medium (DMEM) and [3-(4,5-dimethylthiazol-2-yl)-2,5-diphenyl tetrazolium bromide] (MTT) were purchased from Polyscience Inc. (Warrington, PA, USA). 2-Iminothiolane (Traut's Reagent) was purchased from ThermoFisher Scientific (Waltham, MA, USA). The Enhanced Green Fluorescent Protein (EGFP) encoding plasmid DNA (pDNA) (Promega Co; Madison, WI, USA) was cloned in *Escherichia coli*-DH5α and then was extracted with QIAGEN Mega Plasmid kit according to the manufacturer's protocol.

### Synthesis of dexa-conjugated PPI

The Dexa-conjugated PPIs were synthesized through a two-step reaction.

#### Activation of dexa

The 21-hydroxyl group of Dexa was conjugated with the mesylate group. To this end, Dexa base (210.36 mg, 0.536 mmol) was dissolved in 4 mL of anhydrous pyridine and stirred under continuous argon (Ar) gas stream at 0 °C, and then methanesulfonyl chloride (50.17 µL, 0.644 mmol) was added dropwise to the solution. The reaction was carried out for 5 h, and then 40 mL of ice water was added to stop the reaction. An excess amount of ice water was utilized to wash and filter the white precipitated product via Buchner funnel. Thereafter, to achieve the dry powder of the product (Dexa-mesylate), it was located in a desiccator for an overnight *in vacuo*.

##### The reaction of PPI and dexa-mesylate

PPI (1 equiv.) was dissolved in 1.0 mL of anhydrous DMSO. Dexa-mesylate and Traut’s reagent (both 4 equiv.) were dissolved in 2.0 mL of anhydrous DMSO, and then this solution was slowly added to the PPI solution under Ar gas stream and continuous stirring for 4 h at room temperature. The reaction was halted with cold ethyl acetate. The resultant product was dissolved in double distilled water (ddH_2_O). Then the product was dialyzed against ddH_2_O using dialysis membrane (MWCO 1000) for 48 h. The dialysis medium was refreshed every 12 h. Finally, the product was lyophilized to achieve the Dexa-modified PPI (PPI-Dexa) powder.

### Determining the grafting percentage of dexa on PPI

The percentage of Dexa grafting on primary amine groups of PPI was assessed by determining the amount of free primary amines of the modified PPI G4 and PPI G5 via coupling with TNBS, as described by Snyder and Sobocinski (1975). Briefly, 20 μL of the freshly prepared TNBS solution (15 mg/mL) was added to various amounts of PPIs (dissolved in 600 μL of ddH_2_O) in 96-well microplate. The mixtures were diluted by 200 μL of sodium bicarbonate buffer solution (0.8 M and pH 9.2) and the UV absorbance was measured at 410 nm.

### Evaluating the buffering capacity of prepared PPIs

Various derivatives of PPI G4 and PPI G5 (0.4 mg/mL) were dissolved in ddH_2_O and the pH of solutions was adjusted to 12 using NaOH (1 N). Then HCl (1 N) was added stepwise (5 μL in each step) and the pH change was monitored. The addition of HCl (5 μL) was continued until the pH of solutions had been reached to 2.5. The resultant diagrams, plotting the amount of the added HCl versus the pH of solutions, displayed all the changes in the buffering capacity of the modified PPIs.

### Formation of PPI-pDNA complexes

PPI-pDNA complexes were spontaneously formed by the addition of modified PPIs to pDNA in HEPES-buffered glucose (HBG) [20 mM HEPES in glucose solution (5%)] and further 20 min incubation at room temperature. The complexes with various carriers to pDNA weight ratios (C/P ratio) including 0.5:1, 1:1, 2:1 and 4:1 were provided.

### Size and zeta potential

Zeta potential and size of different polyplexes (at C/P ratio of 2) were determined with Zeta Sizer Nano ZS.

### Gel retardation assay

After a 20 min incubation of PPIs with pDNA in different C/P ratios (0.5:1, 1:1, 2:1 and 4:1), 5 μL of the polyplexes was loaded to agarose gel (1%) and then electrophoresis was carried out for 1 h. The DNA size marker (100-10,000 bp) and the naked pDNA were utilized as a control. Due to tightly compaction, the completely condensed DNA should not move forward through the agarose gel. The pDNA bands were visualized with EtBr.

### Cell culture and in vitro transfection efficiency

Cell culture medium consisted of DMEM, 10% fetal bovine serum, penicillin 100 U/mL, streptomycin 100 μg/mL, was utilized for culturing Neuro2A cells (ATCC CCL-131). All cells were incubated in a humidified atmosphere (more than 90%) containing CO_2_ 5% at 37 °C. Firstly, 1 × 10^4^ Neuro2A cells per well were seeded in 96-well microplate and incubated for 24 h. Next, the medium was replaced with serum- and antibiotic-free DMEM, and then the prepared polyplexes (C/P ratios: 2, 4 and 6) were added and incubated for 4 h. After that, the medium of the cultured cell was refreshed and incubated for an overnight. The cells were lysed with lysis buffer and the transfection yield was measured by fluorescent plate reader and indicated as fluorescence intensity. The excitation and the emission of the expressed EGFP fluorescence were evaluated at 485 and 535 nm wavelengths, respectively.

### Cytotoxicity assay

The cell culture medium condition was the same as the transfection assay. After 24 h incubation, the medium was replaced with serum- and antibiotic-free medium, and then the seeded cells were treated with the same amounts of polyplexes (C/P ratio of 2) which were utilized for *in vitro* transfection assay. After 4 h the medium was refreshed. The metabolic activity of the treated cells was specified with MTT assay 24 h after treatment with the PPI-pDNA complexes as follows: the cells were incubated with MTT solution [10 μL of tetrazolium dye (5 mL/mL) in phosphate-buffered saline (PBS)] for 2 h at 37 °C. After that, the medium was replaced with DMSO (100 μL) and further incubated for 30 min at 37 °C. Finally, the optical absorbance of each well was determined at 630 nm wavelength by a microplate reader (Statfax-2100; Awareness Technology, USA). The metabolic activity of the treated cells was expressed as the percentage of viable cells compared with the untreated control cells which were considered as 100% viable. The results of cytotoxicity assays were expressed as mean ± SD of triplicates.

### Statistical analysis

The statistical analyses were performed using the SPSS software. One-way analysis-of-variance (ANOVA) and Tukey-Kramer tests were performed to analyze the results. The differences between the results of various prepared polyplexes were considered statistically significant when the *p*-value was less than 0.05.

## Results and discussion

### Synthesis of PPI-Dexa

**Figure 1.**
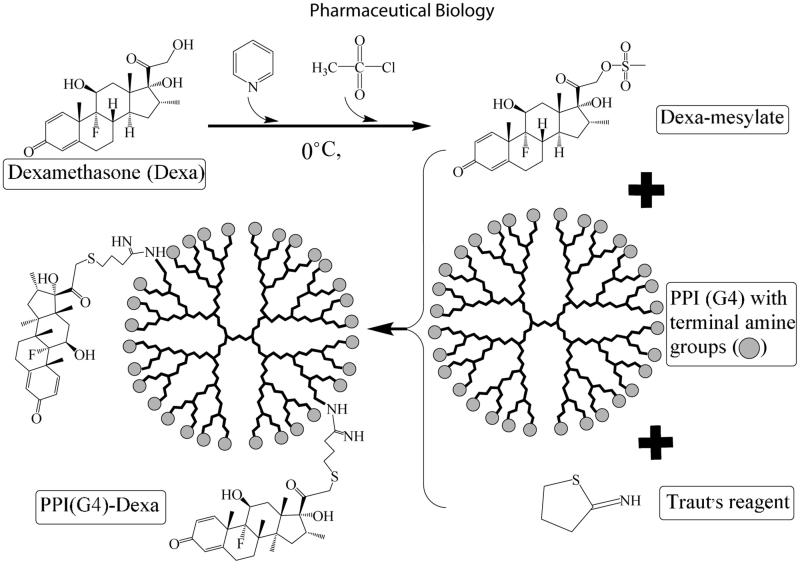
Conjugation of dexamethasone (Dexa) to polypropylenimine (PPI) G4 *via* activation of Dexa with anhydrous pyridine and methanesulfonyl chloride (0 °C), then addition of dexa-mesylate to PPI (G4) in presence of Traut’s reagent.

In the current study, some primary amine groups of PPI were partially substituted with various amounts of Dexa (5, 10 and 20%) through a two-steps reaction (Figure 1). The modified dendrimers in this study were represented as PPIG(X)-Dexa (Y)%, for which X and Y stand the generation of dendrimers and the Dexa grafting percentage, respectively.

### The dexa grafting percentage

**Figure 2.**
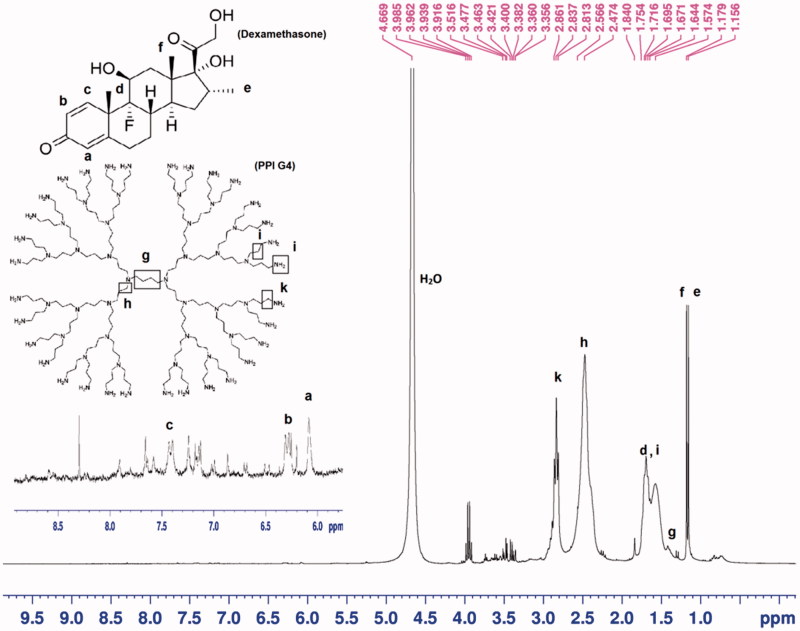
^1^H-NMR of PPI-Dexa.

The actual amount of Dexa grafting to PPI was determined via TNBS assay. Briefly, the standard curves of PPI G4 and PPI G5 were depicted with different concentrations to achieve their standard equations (data not shown). With the calculations based on the absorbance of different concentrations of the Dexa-modified PPIs and the related standard curves, the real Dexa substitution was determined. As indicated in Table 1, the actual degree of Dexa substitution in all of the grafting percentages was less than the initial feed mole percentages. In order to confirm the Dexa substitution in PPIs, the products were analyzed with ^1^H-NMR. As shown in Figure 2, both of the ^1^H-NMR peaks and their related bonds in the schematic molecule structure were labeled with the same lowercase letters.

**Table 1.** The estimated degree of primary amine substitution in the modified PPIs calculated with TNBS assay.

Samples	Initial dexamethasone (Dexa) feed mole %	The calculated substitution of dexamethasone (Dexa) %
PPIG4-Dexa 5%	5	4.84
PPIG4-Dexa 10%	10	8.57
PPIG4-Dexa 20%	20	14.70
PPIG5-Dexa 5%	5	3.89
PPIG5-Dexa 10%	10	8.77
PPIG5-Dexa 20%	20	15.93

### Buffering capacity

The buffering capacity of non-viral vectors is a critical characteristic, influencing the final destination of plasmid DNA. Since non-viral vectors are mainly entered the cells through endocytosis, the escape from endosome, which degrades sensitive NAs, is a determinant step to achieve optimum cell transfection (Habrant et al. 2016). The endosomal escape of PPIs is primarily depended on their proton sponge capability which is influenced by their protonation (buffering) capacity in the acidic pH of endosome (Pan et al. 2013).

**Figure 3.**
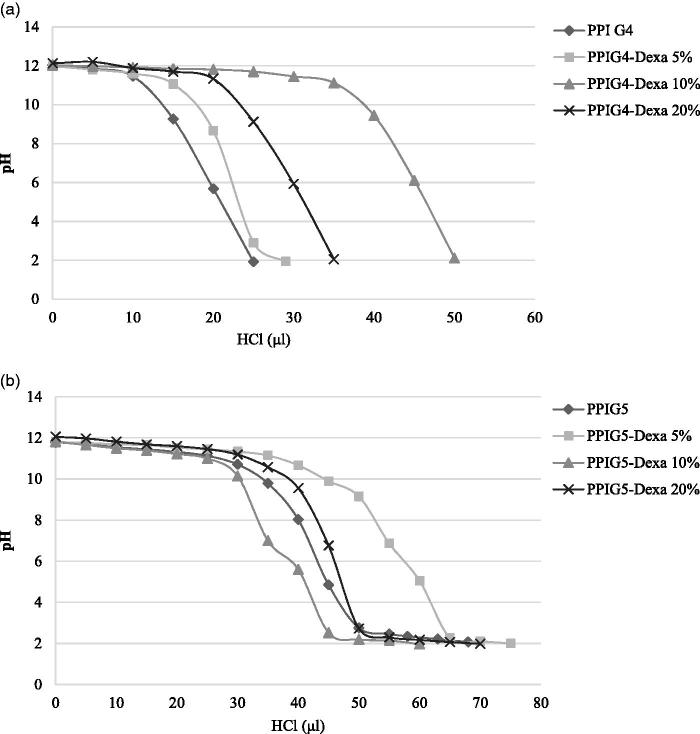
Buffering capacity of Dexa-modified PPI G4 (a) and PPI G5 (b) were illustrated.

As illustrated in Figure 3, the buffering capacity of the Dexa-modified PPIG4s was increased compared with PPI G4. The highest buffering capacity was observed in PPIG4-Dexa 10%, while the 5 and 20% Dexa-modified PPI G4 showed comparable buffering capacity with the unmodified PPI G4. The results of Dexa-modified PPI G5 indicated that the highest buffering capacity was observed in PPIG5-Dexa 5%, while the buffering capacity of PPIG5-Dexa 10% and PPIG5-Dexa 20% were lower and close to PPI G5, respectively. Taking the results of transfection activity into account, it was deduced that the changes of buffering capacity had no significant effect on the transfection efficiency of both Dexa-modified PPI G4 and PPI G5 due in part to the comparable buffering capacity of most Dexa-modified PPIs.

### Size and zeta potential

Size and surface charge of gene carriers are crucial parameters, affecting their cell transfection and toxicity (Kazemi Oskuee et al. 2015). As summarized in Table 2, the size and zeta potential of polyplexes were ranged from 166 to 394 nm and 20 to 27 mV, respectively. With the electrostatic interaction by negatively charged cell surface molecules, the cationic vectors can actively enter the cells (Dehshahri et al. 2012). Thus, the positive charge of gene carriers causes more cellular uptake and more transgene expression. Taking the cytotoxicity of non-viral vectors into account, very high positive charge of gene carriers increases their cytotoxicity which is not favourable. Thus, a moderate positive charge of polyplexes is adequate to actively interact with the cell membrane and enter the cells. One of the major parameters of the non-viral vectors which influence their transfection activity is the size of gene carriers (Khalil et al. 2006). The conjugation of Dexa to PPIs did not significantly change their size. The synthesized polyplexes had acceptable zeta potential as non-viral vectors, while the size of most of them was ranged between 200 nm and 300 nm. Overall, the modified dendrimers had proper physicochemical properties (surface charge and size) for gene delivery.

**Table 2.** Size and zeta potential of polyplexes.

Polyplexes	Size (nm)	Polydispercity index (PDI)	Zeta potential (mV)
PPI G4	284.2 ± 6.7	0.416	26.5 ± 3.9
PPIG4-Dexa 5%	296.0 ± 14.6	0.473	23.3 ± 1.2
PPIG4-Dexa 10%	268.3 ± 6.9	0.423	30.3 ± 2.0
PPIG4-Dexa 20%	166.1 ± 2.5	0.383	20.6 ± 3.2
PPI G5	320.2 ± 18.7	0.468	23.1 ± 2.9
PPIG5-Dexa 5%	316.4 ± 16.8	0.382	26.8 ± 1.0
PPIG5-Dexa 10%	225.7 ± 15.0	0.393	25.8 ± 0.9
PPIG5-Dexa 20%	394.5 ± 15.1	0.490	21.0 ± 1.3

### Gel retardation assay

**Figure 4.**
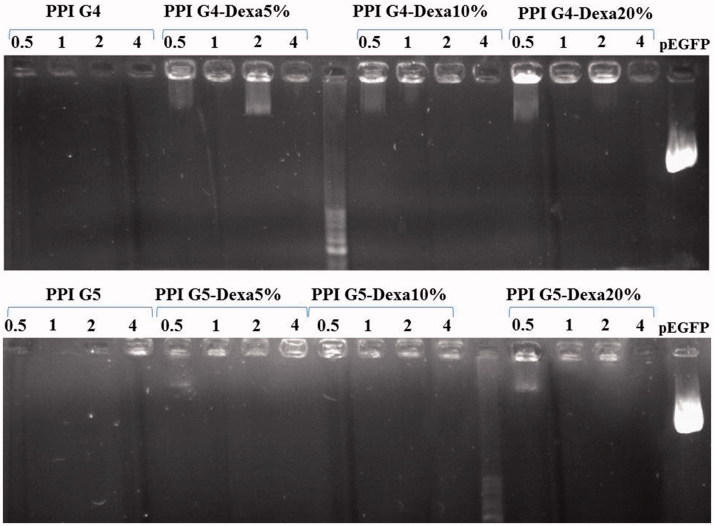
The DNA condensation capability of vectors with various C/P ratios was analyzed with gel retardation assay.

The results of gel retardation assay indicated that all of the modified dendrimers could not completely condense pDNA at C/P ratio of 0.5 whereas, they compacted pDNA more tightly at C/P ratio of one. Irrespective of PPIG4-Dexa 5%, all of the Dexa-modified dendrimers completely compacted pDNA at C/P ratio of two (Figure 4). In addition, the modified dendrimers with C/P ratio of four showed the highest compaction among the vectors tested in the current study. Of note that both of the unmodified PPI G4 and PPI G5 efficiently compacted pDNA even at C/P ratio of 0.5. It is worth noting that the higher DNA condensation capability, the smaller polyplexes will be resulted which are more favourable for gene delivery purposes. On the other hand, the intracellular release of pDNA is of the essence for transgene expression. Thereby, the gene carriers with high C/P ratios might not completely release pDNA in the reducing milieu of cytosol which results in insufficient transgene expression (Dehshahri et al. 2009). Therefore, in order to achieve efficient transfection, a balanced compaction of pDNA is preferred. Inasmuch as the surface charge of vectors determines their DNA condensation capability, the conjugation of adducts (i.e., Dexa) to PPIs decreases their positive charge, which consequentially leads to the better release of pDNA and transfection efficiency provided that their size do not increase.

### Transfection efficiency

**Figure 5.**
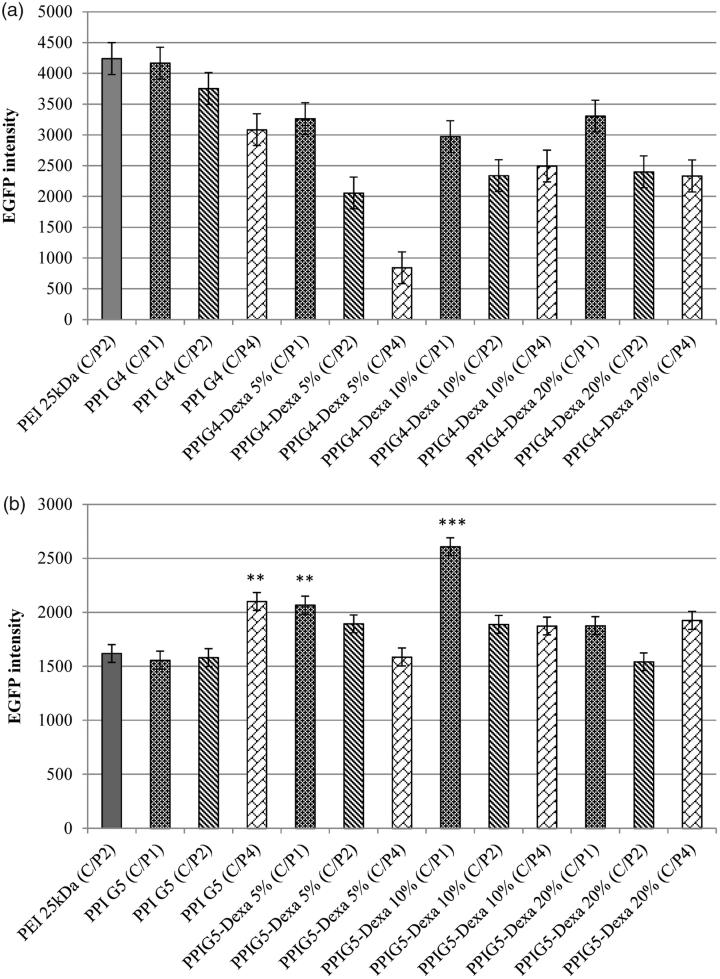
The transfection efficiency of synthesized gene carriers; (a) comparing the cell transfection of forth generation PPIs, and (b) comparing the cell transfection of fifth generation PPIs (**p < 0.01 and ***p < 0.001).

**Figure 6.**
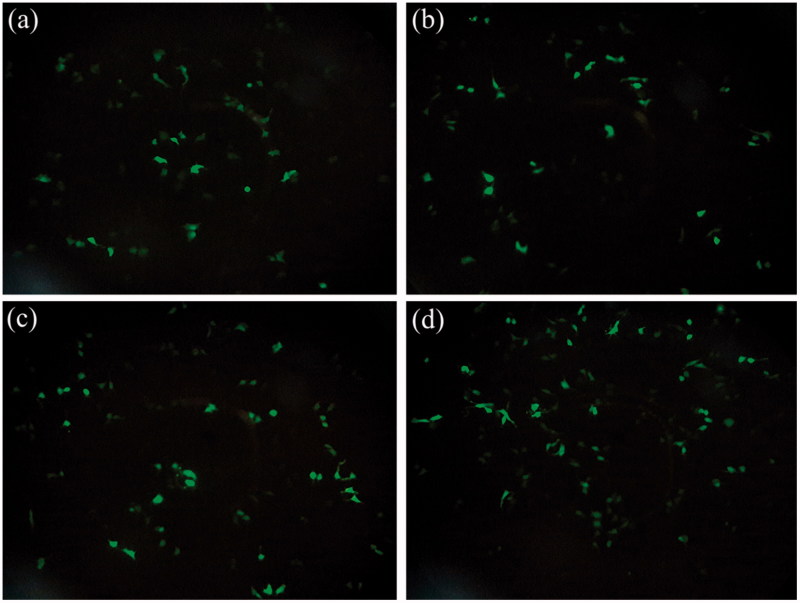
The illustration of EGFP-expressing Neuro2A cells which were transfected with various vectors including: (a) unmodified PPIG4 (C/P ratio of 1), (b) PPIG4-Dexa 5% (C/P ratio of 1), PPIG5-Dexa 5% (C/P ratio of 1) and PPIG5-Dexa 10% (C/P ratio of 1).

In order to evaluate transfection efficiency of prepared gene carriers, all of the vectors were investigated at three C/P ratios including 1, 2 and 4. As illustrated in Figure 5, the transfection efficiency of the Dexa-modified PPIG4s was decreased compared with the unmodified PPIG4s. In addition, their transfection activity was further decreased through the increase of C/P ratios. The Dexa-modified PPIG5s showed better transfection results in comparison with those of PPIG4s. The best transfection potency was observed in PPIG5-Dexa 10% (C/P ratio of 1) which was significantly higher than the unmodified PPIG5 (*p* < 0.001). In consistent with the results of PPIG4s, the transfection activity of the Dexa-modified PPIG5s was not increased through the increase of C/P ratio. At C/P ratio of one, the transfection activity of all of the Dexa-modified PPIG5s was higher than PPIG5. Moreover, at C/P ratio of two, the transfection potency of PPIG5 was increased through the conjugation of Dexa, however, it was not statistically different from that of the unmodified PPIG5. In contrast, at C/P ratio of four, the cell transfection of PPIG5 was decreased through the conjugation of Dexa. PEI 25 kDa, considered as golden standard among non-viral vectors, was utilized to compare cell transfection and toxicity of prepared PPIs with it. The transfection activity of PPIG5 (C/P: 4), PPIG5-Dexa 5% (C/P: 1) and PPIG5-Dexa 10% (C/P: 1) were significantly higher than PEI 25 kDa (Figure 5(b)). Since EGFP was utilized for evaluation of cell transfection, Figure 6 illustrates the fluorescence of Neuro2A cells, transfected with the vectors prepared in this study (Figure 6).

The potential of non-viral vectors in cell transfection is influenced by many factors, the most important of which are those helping to overcome three main aforesaid obstacles of gene delivery (Draghici and Ilies 2015). Thus, the size and buffering capacity of non-viral vectors play crucial roles in their endocytosis and endosomal escape, respectively. Besides the surface charge, the pathway of cellular uptake is mostly determined by the size of gene carriers, in which the vectors smaller than 200 nm enter the cells through clathrin-mediated endocytosis and those with the size between 200 nm and 500 nm enter through caveolae-mediated endocytosis (Rejman et al. 2004). Since the prepared vectors in this study were ranged from 166 nm to 394 nm, it is expected to enter the cell through caveolae-mediated endocytosis which had lower uptake efficiency compared to clathrin-mediated endocytosis. Therefore, the observed transfection results were mainly influenced by their sizes. While the buffering capacity of PPIG5-Dexa 10% was less than PPI G5, it had the smallest size among the Dexa-modified PPIG5s which probably led to its highest transfection efficiency among the unmodified and the Dexa-modified PPIG5s.

### Cytotoxicity assay

**Figure 7.**
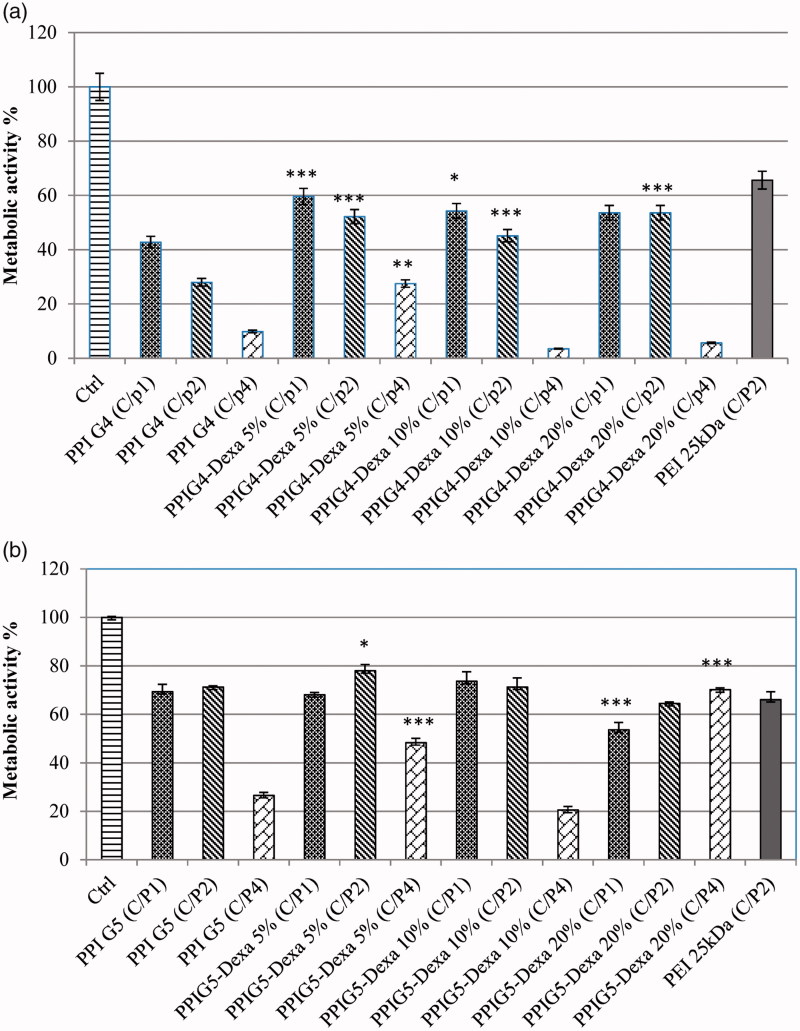
Comparing the cytotoxicity of the Dexa-modified PPIs with the unmodified PPIs in (a) fourth generation PPIs and (b) fifth generation PPIs (*p < 0.05, **p < 0.01 and ***p < 0.001).

In order to determine the cytotoxicity of prepared vectors, MTT assay was utilized. The MTT results of both the Dexa-modified PPIs and the unmodified PPIs indicated an increase in their cytotoxicity through the increase of C/P ratio (Figure 7(a)). The cytotoxicity of polyplexes at C/P ratio of one was significantly decreased through the conjugation of Dexa, among which PPIG4-Dexa 5% showed the highest viability (*p* < 0.001). Also, PPIG4-Dexa 10% had significantly better cytotoxicity profile compared to PPI G4 (*p* < 0.05). Moreover, at C/P ratio of two, all of the Dexa-modified PPIG4s with various grafting percentages showed better cytotoxicity profile in comparison with PPIG4 (*p* < 0.001). Furthermore, at C/P ratio of four, only PPIG4-Dexa 5% showed better cell viability compared to PPIG4 (*p* < 0.01). In comparison with PEI 25 kDa, the cytotoxicity of PPIG4-Dexa 5% at C/P ratio of one was close to it.

The cytotoxicity results of PPIG5s were illustrated in Figure 7(b). The cell toxicity of Dexa-modified PPIG5s at C/P ratio of one indicated that 20% Dexa-substitution in PPIG5 significantly increased their cytotoxicity, while that of PPIG5-Dexa 10% was comparable with the unmodified PPIG5. In addition, at C/P ratio of two, only 5% Dexa-substitution resulted in significant increase of cell viability (*p* < 0.05), while the other grafting percentages resulted in comparable cytotoxicity profile with PPIG5. Moreover, at C/P ratio of four, both of 5% and 20% Dexa-grafting percentages caused a significant increase in cell viability compared with PPIG5 (*p* < 0.001). Furthermore, many of Dexa-modified PPIG5s and unmodified PPIG5s showed comparable cytotoxicity profile with PEI 25 kDa, in which PPIG5, PPIG5-Dexa 5%, PPIG5-Dexa 10% (at C/P ratios of 1 and 2) and PPIG5-Dexa 20% (at C/P ratio of 4) had better cytotoxicity profiles.

The unmethylated CpG motifs of plasmid DNA, loaded to non-viral vectors (PPIs), induce some immunological responses in target cells. Moreover, the non-viral vectors themselves cause some inflammations in the transfected cells. The foregoing phenomena lead to cell transfection decrease, especially through repeated dosing of gene therapy (Tan et al. 1999; Chen Y et al. 2001). Therefore, the decrease of cell inflammation results in better cell transfection. With immunomodulatory and anti-inflammation effects, Dexa is a glucocorticosteroid which was conjugated to PPIs to adjust the cytotoxicity of vectors. Besides the before-mentioned properties, Dexa had been utilized as NLS in non-viral gene carriers (Chen ZZ et al. 2014; Choi et al. 2016; Yoon et al. 2016; Malaekeh-Nikouei et al. 2018). Moreover, with high affinity to its intracellular receptor, Dexa attaches to its cytoplasmic receptor resulting in the transportation of this complex and whatever conjugated to Dexa to the nucleus (Ma et al. 2009). As indicated in Figure 7, irrespective of PPIG5-Dexa 20% (at C/P ratio of 1 and 2), the cytotoxicity of almost all of the Dexa-modified PPIs was improved after the conjugation of Dexa.

## Conclusions

To sum up, the conjugation of Dexa to PPI dendrimers is a promising strategy to improve their transfection potency as well as cytotoxicity as non-viral gene carriers. In this regard, the Dexa-modified PPI (PPIG5-Dexa 10% at C/P ratio of 1) showed promising results. The results of the current study indicated that the physicochemical characteristics of the Dexa-modified PPIs should be optimized to achieve more efficient gene carriers.
